# Prevalence of extra-articular tibia valga morphology in valgus knees and its implications for primary total knee arthroplasty

**DOI:** 10.1186/s13018-022-03418-5

**Published:** 2022-12-09

**Authors:** Salar Sobhi, Riaz J. K. Khan, Daniel P. Fick, Michael Finsterwald

**Affiliations:** 1 Hollywood Medical Centre, The Joint Studio, 85 Monash Avenue, Nedlands, WA 6009 Australia; 2 Hollywood Private Hospital, Monash Avenue, Nedlands, WA 6009 Australia; 3 Faculty of Science and Engineering, Curtin University, Kent Street, Bentley, WA 6102 Australia; 4 School of Medicine, University of Notre Dame, 9 Mouat Street, Fremantle, WA 6959 Australia

**Keywords:** Tibia valga, Pre-operative planning, Total knee arthroplasty, Total knee replacement, Tibial component placement

## Abstract

**Introduction:**

Tibia valga, an extra-articular valgus deformity of the tibia, is common in valgus knees and can result in component misplacement and early total knee arthroplasty (TKA) failure. However, the prevalence and importance of tibia valga in TKA have been seldom reported. This study aims to describe the prevalence and characteristics of tibia valga morphology in valgus knees and describe implications for surgical planning in primary TKA.

**Methods:**

We prospectively examined pre-operative weightbearing whole-body EOS digital radiographs of patients with knee osteoarthritis listed for TKA between December 2018 and December 2020. Hip–knee–ankle angle (HKA), mechanical lateral distal femoral angle (mLDFA), mechanical medial proximal tibial angle (mMPTA), joint line convergence angle (JLCA) and tibial morphology with centre of rotation of angulation of tibia (CORA-tibia) were measured and analysed.

**Results:**

In 830 knees, 253 (30%) and 577 (70%) were classified as valgus and varus, respectively. In valgus knees, 89 knees (35%) had tibia valga. Median CORA-tibia was 2.8° (range 0.2°–10.9°). Tibia valga knees had no difference in mLDFA, higher HKA (5.0^o^ versus 3.0°, *p* = 0.002) and mMPTA (89.6° versus 88.1°, *p* < 0.01), and lower JLCA (2.1° versus 2.3°, *p* < 0.01) compared to non-tibia valga knees. Tibia valga deformity was weakly positively correlated with valgus HKA (*ρ* = 0.23, *p* < 0.001) and mMPTA (*ρ* = 0.38, *p* < 0.001). In varus knees, there were 52 cases of tibia valga (9%) with median CORA-tibia of 3.0° (range 0.5°–5.5°). Tibia valga knees had higher mMPTA (87.0° versus 85.2°, *p* < 0.05) and no difference in HKA, mLDFA and JLCA. CORA-tibia was weakly positively correlated with mMPTA.

**Conclusions:**

Valgus knees may have an extra-articular deformity of the tibia which might be the primary contributor of the overall valgus HKA deformity rather than the distal femoral anatomy. To detect the deformity, full leg-length radiographs should be acquired pre-operatively. Intramedullary instrumentation should be used cautiously in knees with tibia valga when performing TKA.

## Introduction

Coronal alignment of prosthetic components is a critical variable in determining the success of a total knee arthroplasty (TKA) [1, 2, 3, 4]. Achieving proper alignment permits balance of soft tissues and reduced shear and mechanical stresses on the femoral and tibial components as well as degradation of the prosthetic articular surface [4, 5, 6, 7, 8]. Poor alignment of TKA components results in poor functional outcomes and survivorship of the implants which are common in valgus knees [4, 5, 8, 9].

Correction of valgus deformity to neutral alignment remains a challenge [3]. Approximately 10% of patients undergoing total knee arthroplasty (TKA) present with a valgus deformity [10]. The technical challenges associated with correcting a valgus deformity in TKA arise due two main reasons: firstly, a unique combination of bone-tissue and soft-tissue anatomical variations makes it difficult to ensure proper post-surgical alignment; secondly, the lack of adequate pre-operative imaging in the planning phase of TKA which results in less than optimal tibial component placement [2]. The importance of recognising tibial morphology has been previously demonstrated in varus knees where tibial bowing can result in suboptimal tibial cuts in TKA, which increases the risk of varus placement of tibial components [11].

In the literature, it has been historically documented that the majority of knee valgus are primarily due to deformities in the femur, with the conjecture that correction should therefore take place at the femur [12]. However, recently there has been a shift in this theory, suggesting that an isolated deformity in the tibia contributes to the majority of cases with valgus knees [12].

Tibia valga, an extra-articular deformity of the tibia, has been suggested to be a common implicating anatomical variation in valgus knees. Utilisation of intramedullary jigs and tibial stems in valgus knees with such a morphology has been demonstrated to result in malpositioning and cortical impingement and therefore increase the risk of an iatrogenic tibial fracture if pre-operative planning is inadequate [13]. To date, the literature on the subject is limited, with only two studies using full lower-limb weightbearing X-rays examining this morphology in detail despite its potential implications on tibial component placement in TKA [12, 14].

This study aims to describe the prevalence and characteristics of tibia valga morphology in osteoarthritic knees. We also draw implications from these findings to inform and improve pre-operative planning for TKA.

## Methods

This single-centre study protocol was approved by the hospital ethics committee (HREA–SS000359). Patients undergoing primary TKA between December 2018 and December 2020 were drawn from our prospective database and reviewed against the eligibility criteria.

### Inclusion criteria

Patients diagnosed with idiopathic knee osteoarthritis requiring primary TKA by one of the two arthroplasty surgeons (DF and RK) in the above-mentioned timeframe were included. Furthermore, pre-operative imaging of their knees using weightbearing whole body low radiation biplanar EOS digital radiographs had to be available.

### Exclusion criteria

Exclusion criteria included patients with knee osteoarthritis secondary to a traumatic or congenital pathology, history of previous femoral or tibial osteotomy, fracture of the femur or tibia with or without internal fixation, partial arthroplasty of the knee or TKA.

### Radiographic measurements

**Fig. 1:**
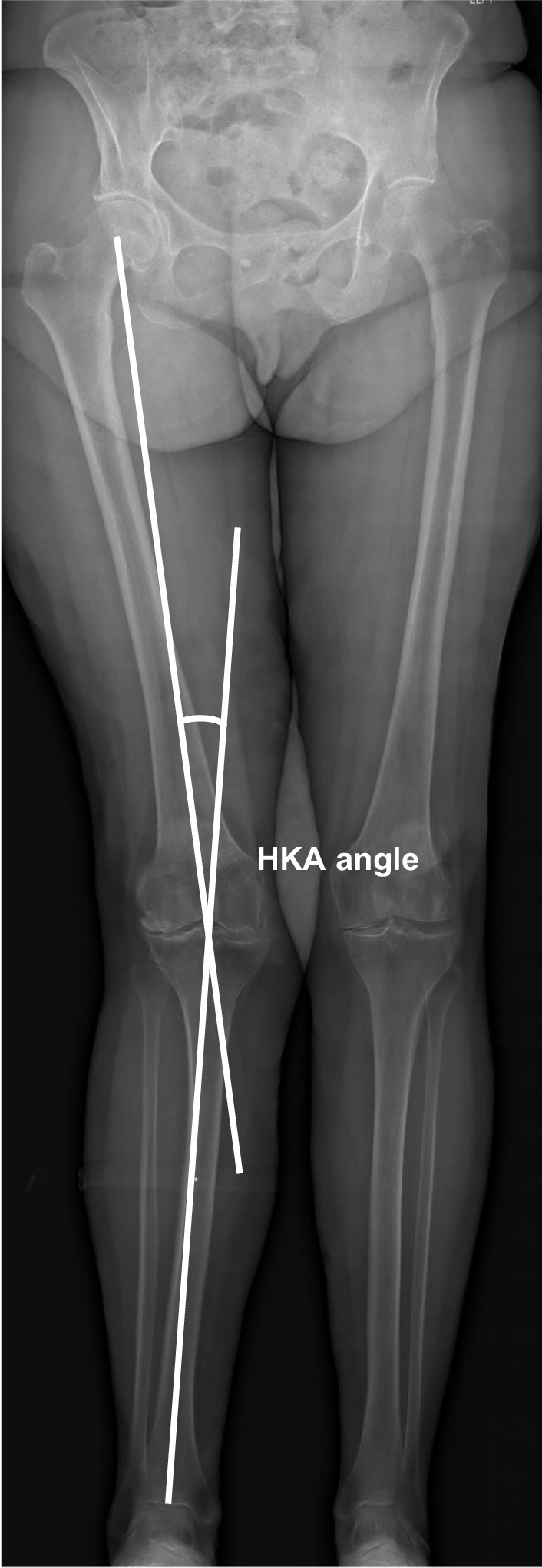
Hip–knee–ankle (HKA) angle. A pre-operative weightbearing lower-limb anteroposterior EOS digital radiograph is shown. The HKA is the angle between the femoral and tibial mechanical axes. Varus, negative; valgus, positive

**Fig. 2:**
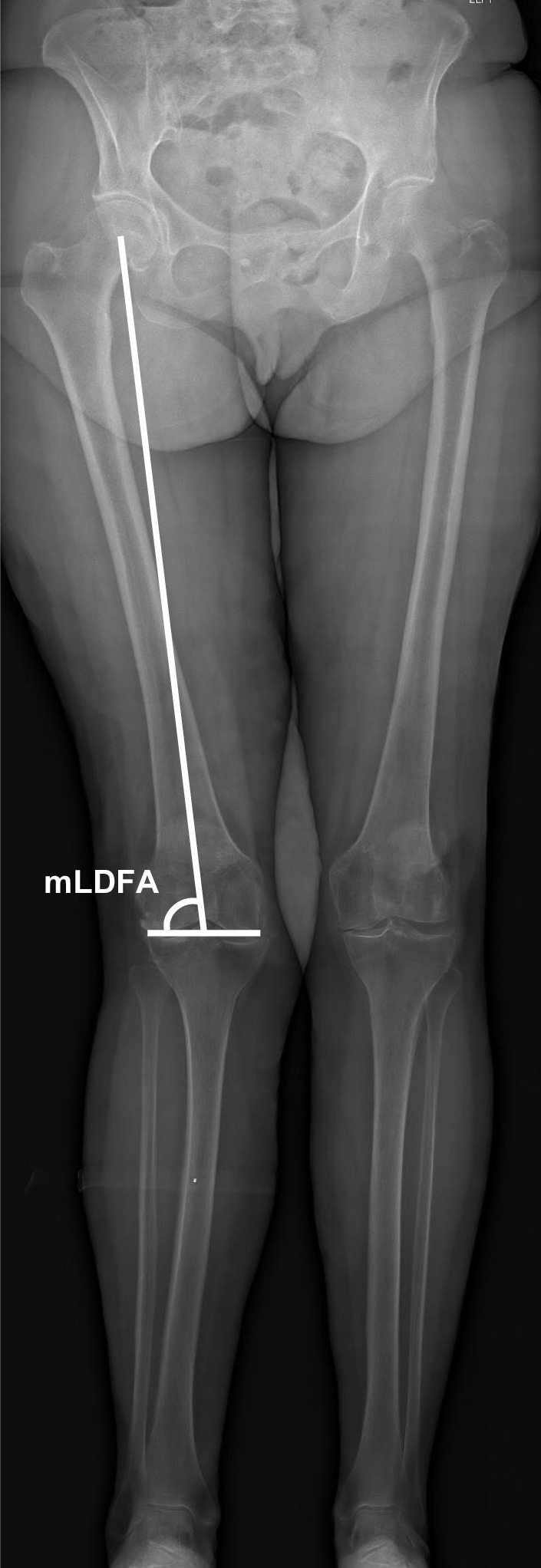
Mechanical lateral distal femoral angle (mLDFA). A pre-operative weightbearing lower-limb anteroposterior EOS digital radiograph is shown. The mLDFA is the angle between the mechanical axis of the femur and its articular surface

**Fig. 3:**
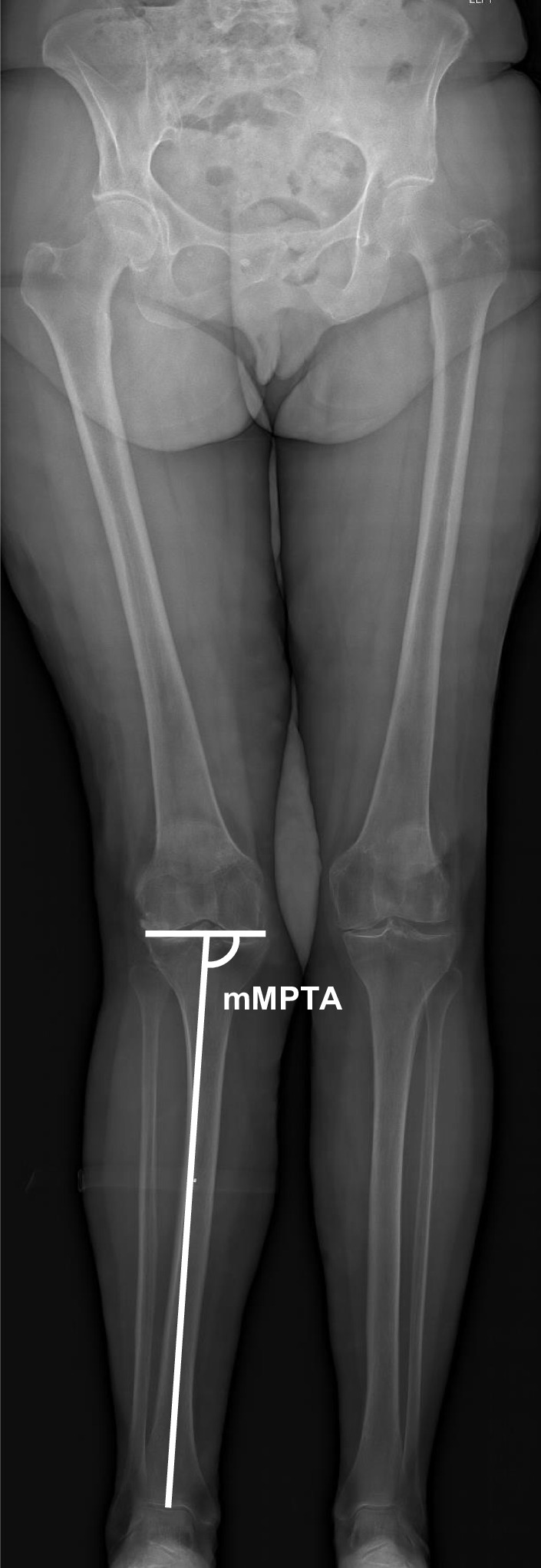
Mechanical medial proximal tibial angle (mMPTA). Pre-operative weightbearing lower-limb anteroposterior EOS digital radiograph is shown. The mLDFA is the angle between the mechanical axis of the femur and its articular surface

**Fig. 4:**
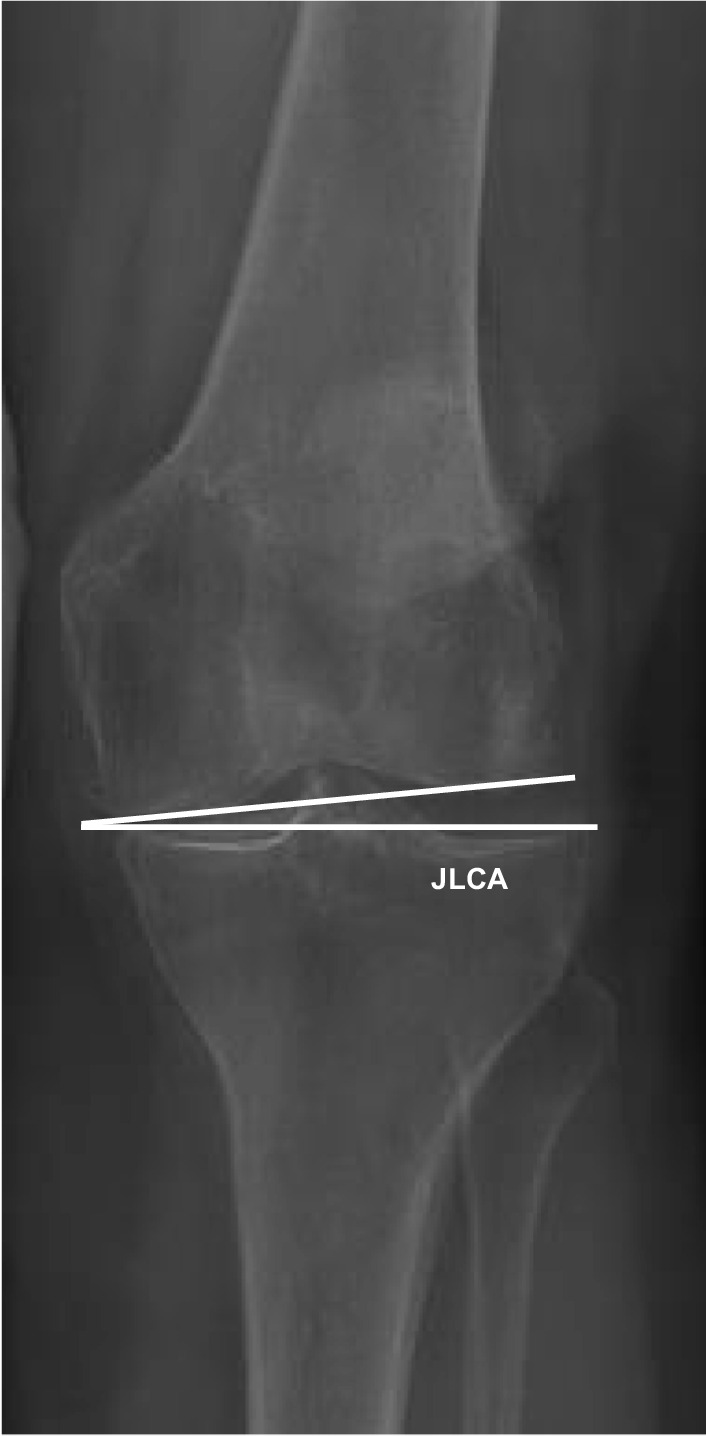
Joint line convergence angle (JLCA). Close-up of the knee joint of a pre-operative, weightbearing lower-limb anteroposterior EOS digital radiograph is shown. The JLCA is the angle formed by the articular surfaces of the femur and tibia

**Fig. 5:**
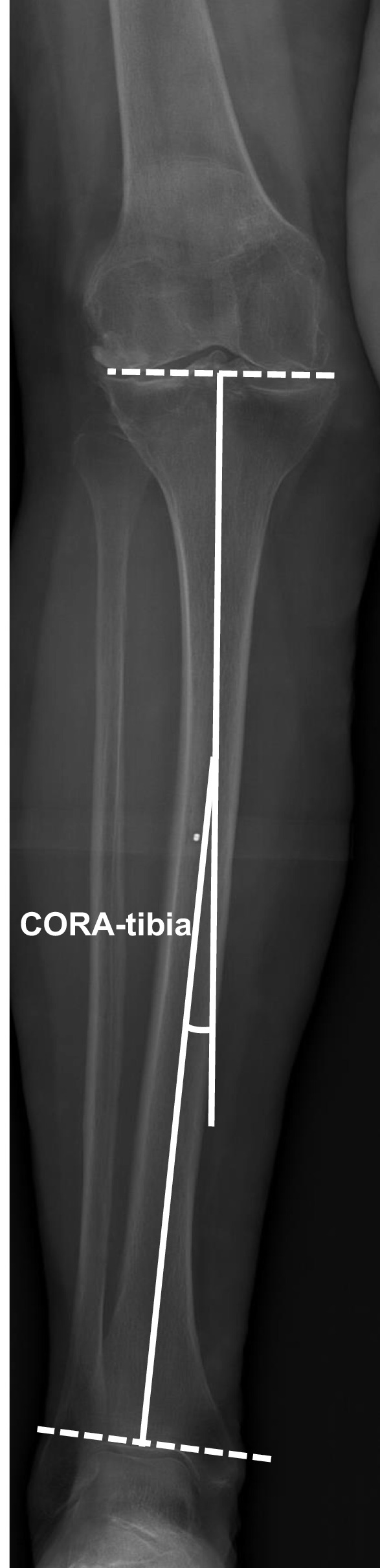
EOS radiograph demonstrating measurement of extra-articular tibial deformity in valgus knee. Close-up of the knee joint and leg of a pre-operative, weightbearing lower-limb anteroposterior EOS digital radiograph is shown. The angle formed by the proximal and distal tibial mechanical axes is the centre of rotation angulation (CORA-tibia)

Digital radiographs were accessed with the hospital’s imaging archiving and communication system (IntelePACS), and measurements were performed with its software (InteleViewer, Intelerad Medical Systems Incorporated, Montreal, Canada). This software contains a ruler, circle and goniometer tools which allows identification of anatomical landmarks to make measurements. Previous studies have demonstrated excellent accuracy, reliability and reproducibility of digital orthopaedic measurements relative to manual modalities [15, 16]. The following pre-operative knee radiographic angles (^o^) were measured to determine the prevalence and characteristics of tibia valga morphology. All measurements were performed by an experienced arthroplasty fellow (MF):

1. *Hip–knee–ankle (HKA) angle*. Used as a measure of lower-limb alignment to determine if the knee joint is either in varus or valgus, defined as the angle between the mechanical axes of the femur and tibia [17]. The mechanical axes of the femur and tibia were defined by a line from cortical centre of the femoral head to the cortical centre of the femoral intercondylar notch and a line from the cortical centre of the talus to the centre of the tibial plateau, respectively (Fig. 1) [18, 19, 20]. In healthy adults, neutral alignment is between 1.0° and 1.5° of varus [17].
2. *Mechanical lateral distal femoral angle (mLDFA)*. Used as a measure of deformity for the distal femur. Defined by the angle between the mechanical axis of the femur described above and the articular surface of the femur (Fig. 2) [18, 19, 20]. In normal knees, the mean mLDFA is 87.5^o^ valgus with a range of 85°–90° [20].
3. *Mechanical medial proximal tibial angle (mMPTA).* A measure of deformity for the proximal tibia which was defined by the angle between the mechanical axis of the tibia described above and the articular surface of the proximal tibia (Fig. 3) [18, 19, 20]. In normal knees, the mean mMPTA is 87.5^o^ varus with a range of 85°–90° [20].
4. *Joint line convergence angle (JLCA).* Measured to evaluate knee joint congruity. Defined as the angle of the two articular surface lines of the distal femur and the proximal tibia. The angle was determined by the femoral condylar joint and the tibial plateau joint line (Fig. 4) [20, 21].
5. *Centre of rotation angulation of proximal tibia (CORA-tibia)*. Used as a measure of extra-articular tibia valga deformity. Defined as the angle formed between the bisection of the proximal and distal mechanical axes of the tibia. The proximal mechanical axis is determined by drawing a line distally from the centre of the tibial plateau bisecting the proximal diaphysis. The distal mechanical axis of the tibia is determined by drawing a line from the centre of the distal articular surface of the tibia proximally bisecting the distal tibial diaphysis. The angle formed by the convergence of these two lines is defined as the CORA-tibia (Fig. 5) [14, 20].

### Statistical analysis

Statistical analyses were performed using R (V4.0.0; R Core Team, Vienna, Austria). Knee radiographs were stratified into valgus and varus knee groups according to their HKA angle measurements and further subdivided as tibia valga or non-tibia valga according to their CORA-tibia measurement. Shapiro–Wilk tests on angular measurements revealed that they were not parametric. Unpaired two-sample Mann–Whitney U tests were performed for univariate analyses and correlation matrices using Spearman’s rho values. *p* < 0.05 was considered statistically significant.

## Results

A total of 830 knees from 520 patients satisfied the criteria for analysis. Measurement of the HKA angle revealed that 253 (30%) and 830 (70%) knees were classified as valgus and varus, respectively. Radiographic characteristics of the valgus and varus knee groups, respectively, are described in Tables 1 and 2.

**Table 1:** Radiographic analysis of valgus knees according to presence of tibia valga

Parameter	Tibia Valga (89/253 = 35%)			Non-Tibia Valga (164/253 = 65%)			*p* value
	Median	*Q*1–*Q*3	Min–Max	Median	*Q*1–*Q*3	Min–Max	
HKA	5.0	2.0–8.0	0.0–14.0	3.0	1.0–6.0	1.0–6.0	**0.002**
mLDFA	85.5	84.1–91.0	79.9–98.0	85.5	84.2–86.8	79.7–97.1	0.97
mMPTA	89.6	88.3–91.0	84.1–94.8	88.1	86.9–89.3	84.0–95.4	** < 0.001**
JLCA	2.1	0.7–2.87	0.0–9.43	2.3	0.9–3.3	0.0–8.8	**0.045**
CORA-tibia	2.8	2.0–3.73	0.23–10.9	0.0	0.0–0.0	0.0–0.0	** < 0.001**

*Q1–Q3* Interquartile range, *HKA* Hip–knee–ankle, *mLDFA* mechanical lateral distal femoral angle (^o^ in valgus), *mMPTA* mechanical medial proximal tibial angle (^o^ in varus), *JLCA* joint line convergence angle, *CORA-tibia* centre of rotation angulation of tibia in valgus. *p <0.05* was considered statistically significant

**Table 2:** Radiographic analysis of varus knees according to presence of tibia valga

Parameter	Tibia Valga (52/577 = 9%)			Non-Tibia Valga (525/577 = 91%)			*p* value
	Median	*Q*1–*Q*3	Min–Max	Median	*Q*1–*Q*3	Min–Max	
HKA	4.0	2.0–7.0	0.0–17.0	5.0	3.0–9.0	0.0–25.0	0.039
mLDFA	87.8	86.4–89.0	83.1–93.9	87.7	86.4–89.0	81.2–103	0.79
mMPTA	87.0	85.5–88.6	81.4–92.6	85.2	83.8–86.7	69.6–94.1	** < 0.001**
JLCA	4.3	2.8–5.8	0.2–10.0	3.9	2.2–5.5	0.0–11.1	0.23
CORA-tibia	− 3.0	− (2.3–3.9)	− (0.5–5.5)	0	0.0–0.0	0.0–4.2	** < 0.001**

*Q1–Q3* Interquartile range, *HKA* Hip–knee–ankle, *mLDFA* mechanical lateral distal femoral angle (^o^ in valgus), *mMPTA* mechanical medial proximal tibial angle (^o^ in varus), *JLCA* joint line convergence angle, *CORA-tibia* centre of rotation angulation of tibia in varus. *p < 0.05* was considered statistically significant

### Valgus knee group radiographic analysis

CORA-tibia measurements revealed that 35% (89 out of 253) of the valgus knees demonstrated a tibia valga morphology. Overall, there was no difference in mLDFA when comparing valgus knees with and without a tibia valga morphology (*p* > 0.05). Valgus knees with tibia valga had greater angular deformity (HKA) compared to non-tibia valga knees (5.0° versus 3.0°, *p* = 0.002) The average mMPTA in valgus knees (89.6°) with tibia valga was within the normal reference range and was found to be significantly higher than those valgus knees without a tibia valga morphology (*p* < 0.001). Convergence of the knee joint (JLCA) was significantly lower in valgus knees with tibia valga compared to those without tibia valga (*p* < 0.05). No valgus knees demonstrated a tibia vara morphology. Furthermore, CORA-tibia was shown to be positively but weakly correlated with HKA (*p* < 0.001) and mMPTA (*p* < 0.001), respectively (Table 3).

**Table 3:** Correlation matrix of knee alignment variables in valgus knees with tibia valga

		HKA	mLDFA	mMPTA	JLCA	CORA-tibia
HKA	Spearman’s rho	–	–	–	–	–
	*p *value	–	–	–	–	–
mLDFA	Spearman’s rho	**−0.4208**	–	–	–	–
	*p *value	** < 0.001**	–	–	–	–
mMPTA	Spearman’s rho	**0.2633**	−0.0063	–	–	–
	*p *value	** < 0.001**	0.920	–	–	–
JLCA	Spearman’s rho	**0.3565**	**−0.1438**	−0.0489	–	–
	*p *value	** < 0.001**	**0.022**	0.438	–	–
CORA-tibia	Spearman’s rho	**0.2278**	0.0017	**0.3812**	0.135	–
	*p *value	** < 0 .001**	0.979	** < 0.001**	0.592	–

*HKA* Hip–knee–ankle, *mLDFA* mechanical lateral distal femoral angle, *mMPTA* mechanical medial proximal tibial angle, *JLCA* joint line convergence angle, *CORA-tibia* centre of rotation angulation of tibia in valgus. *p < 0.05* was considered statistically significant

### Varus knee group radiographic analysis

CORA-tibia measurements revealed that 9% (52 out of 577) varus knees demonstrated a tibia valga morphology. There was no difference in mLDFA and JLCA when comparing varus knees with and without a tibia valga morphology (*p* > 0.05). Varus knees with tibia valga had a lower HKA compared to non-tibia valga knees (4.0^o^ versus 5.0°, *p* = 0.039). Furthermore, the average mMPTA in varus knee with tibia valga was found to be significantly higher than those varus knees without a tibia valga morphology although the mMPTA in these knees was within the normal reference range (*p* < 0.05). In this group, CORA-tibia was shown to be weakly positively correlated with mMPTA (*p* < 0.05) (Table 4).

**Table 4:** Correlation matrix of knee alignment variables in varus knees with tibia valga

		HKA	mLDFA	mMPTA	JLCA	CORA-tibia
HKA	Spearman’s rho	–	–	–	–	–
	*p *value	–	–	–	–	–
mLDFA	Spearman’s rho	**0.3284**	–	–	–	–
	*p *value	** < 0.001**	–	–	–	–
mMPTA	Spearman’s rho	−**0.5990**	–0.0167	–	–	–
	*p *value	** < 0.001**	0.690	–	–	–
JLCA	Spearman’s rho	**0.5932**	0.0526	−**0.2046**	–	–
	*p *value	** < 0.001**	0.208	** < .001**	–	–
CORA-tibia	Spearman’s rho	−0.0412	−0.0056	**0.1185**	0.0721	–
	*p *value	0.325	0.894	**0.004**	0.084	–

*HKA* Hip–knee–ankle, *mLDFA* mechanical lateral distal femoral angle, *mMPTA* mechanical medial proximal tibial angle, *JLCA* joint line convergence angle, *CORA-tibia* centre of rotation angulation of tibia in valgus. *p < 0.05* was considered statistically significant

## Discussion

Our study illustrates the prevalence and characteristics of tibia valga in a cohort of patients with knee osteoarthritis using EOS imaging technology. Firstly, we showed that tibia valga morphology is common in valgus knees, with 35% of knees demonstrating this anatomical variant. Secondly, we showed that the distal femur in valgus knees with tibia valga is within the normal anatomical range. Thirdly, we demonstrated that the degree of tibia valga is positively correlated to the overall angular deformity of patients with valgus knees. This study highlights that valgus knees may have an extra-articular deformity of the tibia which might be the primary contributor of the overall valgus HKA deformity rather than the distal femoral anatomy.

The results of our study are consistent with the only two other studies [13, 14] which have radiographically analysed tibia valga morphology in valgus knees. Alghamdi et al.’s [14] retrospective review of 97 osteoarthritic valgus knees prior to TKA via full lower-limb weightbearing X-rays demonstrated a similar prevalence of tibia valga to ours, with 53% of valgus knees illustrating a tibia valga. Furthermore, they also demonstrated that the extra-articular tibia valga deformity was an isolated deformity, with mLDFA reported to be within normal limits and contributed significantly to the overall mechanical femorotibial alignment [14, 20]. This is also consistent with Eberbach et al.’s [19] study challenging the previously accepted dogma that the primary deformity of valgus knees originates in the femur. Our results not only demonstrate the characteristics of tibia valga morphology, but we also demonstrate that there is a positive correlation between the magnitude of the deformity (CORA-tibia) and the overall HKA valgus deformity of the knee.

**Fig. 6:**
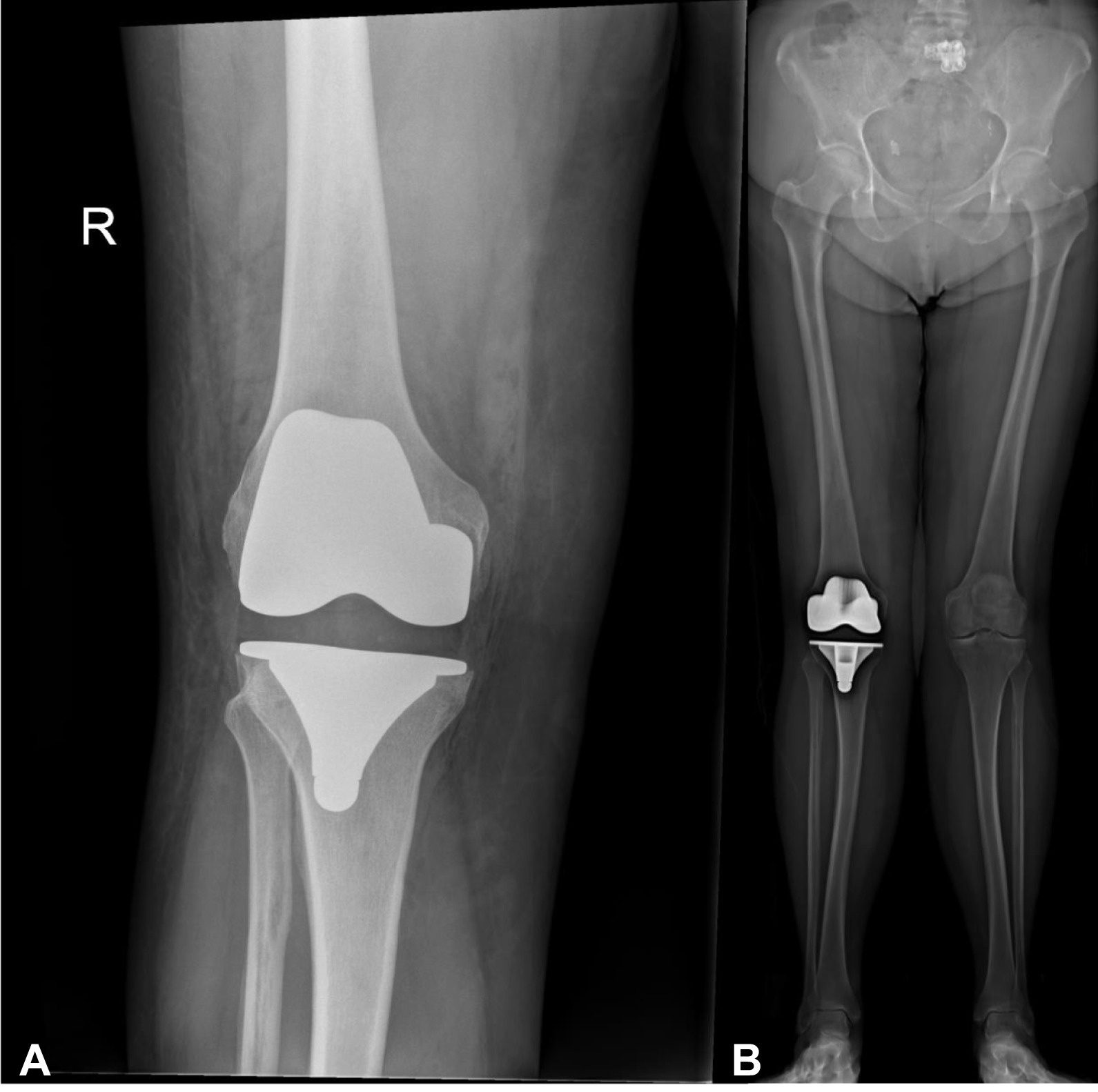
Clinical significance of full lower-limb weightbearing radiographs in total knee arthroplasty (TKA). **A** Post-TKA anteroposterior radiograph of the right knee demonstrating satisfactory position. **B** Same patient with a post-TKA weightbearing lower-limb anteroposterior EOS digital radiograph, demonstrating an extra-articular tibia valga deformity and post-operative valgus alignment of the right lower limb.

Our results demonstrate the importance of screening for tibia valga as part of pre-operative planning as it can have implications for the TKA procedure. Restoring the mechanical axis of the lower limb and ensuring accurate cuts of the femur and tibia are critical in determining the success and survivorship of a TKA [22]. Restoration of the mechanical axis is achieved through bone cuts perpendicular to the mechanical axes of the femur and tibia, respectively. It has been previously demonstrated that usual tolerance of error for bone cuts is approximately 3° from its ideal position [23]. The correct placement of cutting jigs and alignment guides are critical in ensuring accurate placement of femoral and tibial components in TKA [24, 25]. Although intra-articular causes of valgus deformities can be managed intra-operatively, extra-articular deformities of the lower limb may be overlooked both clinically and surgically. Figure 6 illustrates such an example, where tibia valga can be overlooked if only knee X-rays were available for operative planning. Given the small window for error for component placement in TKA means that presence of a tibia valga deformity can significantly mislead the orthopaedic surgeon in their tibial cuts [23]. Therefore, we recommend that the surgeon adequately plans their TKA with pre-operative lower-limb weightbearing radiographs to screen for extra-articular deformities which may contribute to the angular deformity of the knee, given their common prevalence. Previous studies have demonstrated the benefit of standing lower-limb films compared to short-leg films which many orthopaedic surgeons still utilise in the pre-operative planning phase, with their greater accuracy in determining overall tibiofemoral alignment [26, 27, 28].

Furthermore, the common prevalence of tibia valga morphology in valgus knees suggests implications regarding the optimal approach for tibial component placement in TKA. On average in non-arthritic knees, the articular surface of the tibia is 3° varus to its mechanical axis [13]. However studies have demonstrated that tibial component placement in TKA should be 90° to the mechanical axis as this allows the prosthetic component to evenly distribute its weight across the tibia and therefore prolong the survivorship of the implant [14, 25, 29]. The literature has differing evidence regarding the use of intramedullary and extramedullary tibial cutting guides in TKA. Although Reed et al.’s [30] randomised-controlled trial comparing intramedullary and extramedullary cutting guides for tibial resection in 135 TKAs demonstrated that intramedullary cutting guides resulted in a significantly better tibial alignment compared to extramedullary cutting guides, Ko et al. [31] and Yau et al. [32] demonstrated that intramedullary cutting guides in varus knees performed poorly with up to 30% of patients receiving unacceptable tibial cuts. Additionally, Dennis et al. [33] demonstrated that the use of extramedullary cutting guides in TKA resulted in better tibial component alignment compared to intramedullary cutting guides (88% versus 72%). Given the increased likelihood of cortical impingement of the intramedullary cutting guide which can result in less accurate tibial cuts in a valgus knee with significant tibia valga, we suggest the utilisation of extramedullary cutting guides in these knees. Palanisami et al.’s [13] study reaffirms this suggestion, demonstrating that excellent prosthetic component placement can be achieved in valgus knees with tibia valga using extramedullary cutting guides. Similarly, the identification of extra-articular tibia valga in the pre-operative planning of revision TKA is critical, as the use of a straight stem for the tibial component might abut the cortex; hence, offset options need to be available intra-operatively. Therefore, we recommend post-TKA lower-limb weightbearing radiographs to ensure adequate component placement, especially in cases of tibia valga where tibial component placement may appear in varus relative to the proximal tibia.

### Limitations

This study must be viewed in consideration of its limitations. We included all patients with EOS scans, irrespective of rotation, therefore scans where the patella was not visible over the femoral condyles where included. This may reduce the accuracy of the geometric values which were obtained in this study; however, we used an experienced arthroplasty fellow to ensure the most consistent and reproducible measurements were made. Secondly, given the cross-sectional nature of this study, it does not highlight the aetiology and progression of tibia valga (for example, if it is due to lateral compartment knee osteoarthritis or a congenital or developmental deformity). Furthermore, our results demonstrated that tibia valga is also an uncommon occurrence in varus knees. What we know from the literature is that when lower-limb alignment changes, the distribution of stress forces on the tibia can change and result in bone remodelling which may account for this observation [34]. Therefore, longitudinal radiographic studies of patients experiencing knee osteoarthritis can help to address this gap in the literature.

## Conclusions

Tibia valga is a common anatomical morphology occurring in 35% of patients with valgus knee osteoarthritis, whereas it is uncommon in varus knees. This study highlights that valgus knees may have an extra-articular deformity of the tibia which might be the primary contributor of the overall valgus HKA deformity rather than the distal femoral anatomy. To detect the deformity, full leg-length radiographs should be acquired pre-operatively. Intramedullary instrumentation should be used with caution in knees with significant tibia valga when performing TKA.

## Data Availability

The datasets used and/or analysed during the current study are available from the corresponding author on reasonable request.

## References

[1] Schiraldi M, Bonzanini G, Chirillo D, de Tullio V (2016). Mechanical and kinematic alignment in total knee arthroplasty. Ann Transl Med.

[2] Rossi R, Rosso F, Cottino U, Dettoni F, Bonasia DE, Bruzzone M (2014). Total knee arthroplasty in the valgus knee. Int Orthop.

[3] Cerciello S, Lustig S, Servien E, Batailler C, Neyret P (2017). Correction of tibial valgus deformity. J Knee Surg.

[4] D’Lima DD, Hermida JC, Chen PC, Colwell CW Jr. Polyethylene wear and variations in knee kinematics. Clin Orthop Relat Res^®^. 2001;392:124–30. 10.1097/00003086-200111000-00015 11716373

[5] Cherian JJ, Kapadia BH, Banerjee S, Jauregui JJ, Issa K, Mont MA (2014). Mechanical, anatomical, and kinematic axis in TKA: concepts and practical applications. Curr Rev Musculoskelet Med.

[6] Oswald MH, Jakob RP, Schneider E, Hoogewoud H-M (1993). Radiological analysis of normal axial alignment of femur and tibia in view of total knee arthroplasty. J Arthroplasty.

[7] Ensini A, Catani F, Leardini A, Romagnoli M, Giannini S. Alignments and clinical results in conventional and navigated total knee arthroplasty. Clin Orthop Relat Res^®^. 2007;457:156–62. 10.1097/BLO.0b013e3180316c92 17195810

[8] Berend ME, Ritter MA, Meding JB, Faris PM, Keating EM, Redelman R, et al. The Chetranjan Ranawat Award: tibial component failure mechanisms in total knee arthroplasty. Clin Orthop Relat Res^®^. 2004;428:26–34. 10.1097/01.blo.0000148578.22729.0e 15534515

[9] Sikorski J (2008). Alignment in total knee replacement. J Bone Jt Surg.

[10] Ranawat AS, Ranawat CS, Elkus M, Rasquinha VJ, Rossi R, Babhulkar S (2005). Total knee arthroplasty for severe valgus deformity. J Bone Jt Surg Ser A..

[11] Chiu CC, Huang CL, Weng SF, Sun LM, Chang YL, Tsai FC (2011). A multidisciplinary diabetic foot ulcer treatment programme significantly improved the outcome in patients with infected diabetic foot ulcers. J Plast Reconstr Aesthet Surg.

[12] Marin Morales LA, Gomez Navalon LA, Zorrilla Ribot P, Salido Valle JA (2000). Treatment of osteoarthritis of the knee with valgus deformity by means of varus osteotomy. Acta Orthop Bel.

[13] Palanisami D, George MJ, Hussain AM, Chunchesh MD, Natesan R, Shanmuganathan R (2019). Tibial bowing and tibial component placement in primary total knee arthroplasty in valgus knees: are we overlooking?. J Orthop Surg.

[14] Alghamdi A, Rahme M, Lavigne M, Masse V, Vendittoli PA (2014). Tibia valga morphology in osteoarthritic knees: importance of preoperative full limb radiographs in total knee arthroplasty. J Arthroplasty.

[15] Prakash U, Wigderowitz C, McGurty D, Rowley D (2001). Computerised measurement of tibiofemoral alignment. J Bone Jt Surg.

[16] Rozzanigo U, Pizzoli A, Minari C, Caudana R (2005). Alignment and articular orientation of lower limbs: manual vs computer-aided measurements on digital radiograms. Radiol Med (Torino).

[17] Sheehy L, Felson D, Zhang Y, Niu J, Lam Y-M, Segal N (2011). Does measurement of the anatomic axis consistently predict hip-knee-ankle angle (HKA) for knee alignment studies in osteoarthritis? analysis of long limb radiographs from the multicenter osteoarthritis (MOST) study. Osteoarthr Cartil.

[18] Durandet A, Ricci P-L, Saveh AH, Vanat Q, Wang B, Esat I, et al. Radiographic analysis of lower limb axial alignments. In: Proceedings of the world congress on engineering p. 3–5

[19] Eberbach H, Mehl J, Feucht MJ, Bode G, Südkamp NP, Niemeyer P (2017). Geometry of the valgus knee: contradicting the dogma of a femoral-based deformity. Am J Sports Med.

[20] Paley D, Herzenberg JE, Tetsworth K, McKie J, Bhave A (1994). Deformity planning for frontal and sagittal plane corrective osteotomies. Orthop Clin North Am.

[21] Higuchi T, Koseki H, Yonekura A, Chiba K, Nakazoe Y, Sunagawa S (2019). Comparison of radiological features of high tibial osteotomy and tibial condylar valgus osteotomy. BMC Musculoskelet Disord.

[22] Barrett WP, Mason JB, Moskal JT, Dalury DF, Oliashirazi A, Fisher DA (2011). Comparison of radiographic alignment of imageless computer-assisted surgery vs conventional instrumentation in primary total knee arthroplasty. J Arthroplasty.

[23] Chiu K, Yau W, Ng T, Tang W (2008). The accuracy of extramedullary guides for tibial component placement in total knee arthroplasty. Int Orthop.

[24] Luo C-F (2004). Reference axes for reconstruction of the knee. Knee.

[25] Bargren JH, Blaha J, Freeman M (1983). Alignment in total knee arthroplasty. Correlated biomechanical and clinical observations. Clin Orthop Relat Res.

[26] Petersen TL, Engh GA (1988). Radiographic assessment of knee alignment after total knee arthroplasty. J Arthroplasty.

[27] Morgan SS, Bonshahi A, Pradhan N, Gregory A, Gambhir A, Porter M (2008). The influence of postoperative coronal alignment on revision surgery in total knee arthroplasty. Int Orthop.

[28] Skyttä ET, Haapamäki V, Koivikko M, Huhtala H, Remes V (2011). Reliability of the hip-to-ankle radiograph in determining the knee and implant alignment after total knee arthroplasty. Acta Orthop Belg.

[29] Heyse TJ, Decking R, Davis J, Boettner F, Laskin RS (2009). Varus gonarthrosis predisposes to varus malalignment in TKA. HSS J.

[30] Reed M, Bliss W, Sher J, Emmerson K, Jones S, Partington P (2002). Extramedullary or intramedullary tibial alignment guides: a randomised, prospective trial of radiological alignment. J Bone Jt Surg.

[31] Ko P, Tio M, Ban C, Mak Y, Ip F, Lam J (2001). Radiologic analysis of the tibial intramedullary canal in Chinese varus knees: implications in total knee arthroplasty. J Arthroplasty.

[32] Yau W, Chiu K, Tang W, Ng T (2007). Coronal bowing of the femur and tibia in Chinese: its incidence and effects on total knee arthroplasty planning. J Orthop Surg.

[33] Dennis DA, Channer M, Susman MH, Stringer EA (1993). Intramedullary versus extramedullary tibial alignment systems in total knee arthroplasty. J Arthroplasty.

[34] Hunter DJ, Zhang Y, Niu J, Goggins J, Amin S, LaValley MP (2006). Increase in bone marrow lesions associated with cartilage loss: a longitudinal magnetic resonance imaging study of knee osteoarthritis. Arthritis Rheum Off J Am Coll Rheumatol.

